# Association of Baseline Neutrophil-to-Lymphocyte Ratio with Clinicopathological Characteristics of Papillary Thyroid Carcinoma

**DOI:** 10.1155/2017/8471235

**Published:** 2017-05-09

**Authors:** Dimitrios K. Manatakis, Sofia Tseleni-Balafouta, Dimitrios Balalis, Vasiliki N. Soulou, Dimitrios P. Korkolis, George H. Sakorafas, Georgios Plataniotis, Emmanouil Gontikakis

**Affiliations:** ^1^First Department of Surgical Oncology, St. Savvas Cancer Hospital, Athens, Greece; ^2^Department of Pathology, School of Medicine, National and Kapodistrian University of Athens, Athens, Greece

## Abstract

**Objective:**

To investigate the potential association of neutrophil-to-lymphocyte ratio (NLR), a surrogate systemic inflammatory biomarker, with clinical and pathological characteristics of papillary thyroid cancers.

**Methods:**

205 patients with papillary carcinoma were identified from the institutional thyroid cancer database between 2006 and 2015 (55 males, 150 females, mean age 51.2 ± 14.7 years). NLR was calculated as the absolute neutrophil count divided by the absolute lymphocyte count, based on the preoperative complete blood cell counts.

**Results:**

NLR was significantly higher in carcinomas with extrathyroidal invasion (2.74 ± 01.24 versus 2.39 ± 0.96, *p* = 0.04) and bilateral (2.67 ± 1.15 versus 2.35 ± 0.96, *p* = 0.03) and multifocal tumours (2.65 ± 1.08 versus 2.29 ± 0.96, *p* = 0.01), as well as lymph node-positive tumours (3.12 ± 1.07 versus 2.41 ± 1.02, *p* = 0.03). On the other hand, NLR values were not associated with gender, age, tumour size, histologic subtype, the presence of thyroiditis, and TNM staging.

**Conclusions:**

As an index of inflammation, NLR is inexpensive, readily available, and easy to extract from routine blood tests. We found increased NLR values in papillary carcinomas with poorer histopathological profile and more aggressive clinical behaviour. Whether this systemic inflammatory response, as expressed by the NLR, represents the inflammatory microenvironment leading to tumourigenesis, or is a tumour-associated phenomenon, remains to be elucidated and warrants further study.

## 1. Introduction

Tumourigenesis is the result of dysregulation of a delicate balance between elaborate cancer-promoting and cancer-inhibiting molecular pathways. Inflammation plays a critical role in all aspects of cancer, including development, progression, and metastasis, and this complex interplay between neoplastic cells and their inflammatory microenvironment may have significant influence on patient prognosis [1]. Inflammatory cells have probably a protean dual role, pro- and anticarcinogenic, depending on the circumstances of tumour/host interactions. This missing link between inflammation and tumour biology and course remains to be elucidated.

Papillary thyroid carcinoma (PTC), the most common endocrine malignancy, exhibits a close association with chronic inflammation; however, recent studies have produced inconsistent results concerning inflammation and tumour behaviour. An increased incidence of papillary carcinomas is observed in patients with thyroiditis, although chronic lymphocytic thyroiditis may ultimately correlate with improved prognosis in patients with PTC [2].

Several studies to date have shown that inflammatory biomarkers (CRP, TNFa, IL-6, neutrophil-to-lymphocyte ratio) reliably predict poor prognosis in a variety of malignant neoplasms (oesophageal, gastric, pancreatic, colonic, ovarian, renal, and lung) [1–3]. As a surrogate inflammatory marker, neutrophil-to-lymphocyte ratio (NLR) is readily available and easy to extract from routine blood tests. However, its use in the preoperative assessment and postoperative follow-up of PTC cases is still limited and controversial.

Initial studies reported higher NLR in larger tumours and older patients, and generally higher NLR predicted higher risk of recurrence and poor prognosis [3–5]. Other investigators however failed to reproduce these results [6, 7]. Moreover, wide variations in proposed cutoff values contribute to this ongoing debate [5, 8].

The aim of our study was to evaluate a possible association of preoperative NLR with clinicopathological characteristics of patients with PTC, in order to determine its status as a potential biomarker in the pretreatment setting.

## 2. Materials and Methods

Patients diagnosed with PTC between January 2006 and December 2015 at the First Department of Surgical Oncology, St. Savvas Cancer Hospital, were retrospectively identified from the institutional thyroid surgery database. Demographic characteristics (age, sex, and comorbidity), laboratory test results, and pathology outcomes were documented for each patient.

Included in the analysis were all consecutive patients, aged ≥18 years, who underwent thyroidectomy for papillary carcinoma (>1.1 cm) or microcarcinoma (≤1.0 cm), either suspected by the preoperative FNA biopsy or confirmed in the final histopathology. Excluded were patients with known haematological disorders, chronic medical conditions affecting white blood cell counts, past history of malignancy, acute myocardial infarction or coronary revascularisation within 6 months before surgery, and glucocorticoid administration within 3 months before surgery. Noneuthyroid patients, patients with acute infections or with baseline total WBC count outside the institutional reference range (4000–10,000/ml), were also excluded.

Neutrophil-to-lymphocyte ratio was calculated as the absolute neutrophil count divided by the absolute lymphocyte count, based on the preoperative complete blood cell count. According to our preoperative assessment protocol, fasting baseline blood samples are routinely obtained between 08:00 and 10:00 am on the day before surgery and include haematocrit, haemoglobin, total WBC and automated differential counts (neutrophils, lymphocytes, monocytes, basophils, and eosinophils) and platelets. This standardised protocol contributed to adjusting for the known impact of circulating hormones (circadian rhythm) on the number and distribution of WBC subtypes.

Pathology reports were reviewed independently by two authors to determine thyroid specimen weight, tumour size, uni- or multifocality, uni- or bilaterality, lymph node metastasis, extrathyroidal invasion, TNM stage according to AJCC 7th edition, presence of thyroiditis, and PTC histologic subtype (classic versus follicular variant). Other variants (diffuse sclerosing, tall cell, columnar, oncocytic, solid, clear cell, cribriform morular, hobnail, and dedifferentiated) were much more rarely encountered and therefore precluded any meaningful comparisons. Patient subgroups based on clinicopathological parameters were compared in terms of total WBC, neutrophil, and lymphocyte counts as well as NLR values. Tumour size was determined according to the greatest diameter of the lesion based on the postoperative histopathology report. Multifocal tumours were defined as 2 or more foci of PTC in the surgical specimen. Bilateral tumours were defined as PTC lesions affecting both thyroid lobes, in contrast to unilateral tumours, in which lesions were found unilaterally. Finally, thyroiditis was identified by lymphocytic infiltration and formation of lymph follicles and included both cases of Hashimoto's and nonspecific lymphocytic thyroiditis.

On the basis of NLR stratification, patients were subsequently divided into two subgroups by the median NLR value, the lymphophilic low NLR and the neutrophilic high NLR groups. These groups were compared in terms of the aforementioned clinicopathological characteristics.

Continuous variables were expressed as mean ± standard deviation, while categorical variables were expressed as frequencies or percentages. Statistical analysis was performed on SPSS, version 20.0, using Student's *t*-test and ANOVA for continuous variables and chi-square test for categorical variables. Statistical significance was set to *p* < 0.05. The study was approved by the institutional research ethics committee.

## 3. Results

During the course of the study period, 233 patients were diagnosed with PTC. Of those, 205 patients fulfilled the inclusion criteria and were included in the final analysis. Demographic characteristics and haematological data are shown in Table 1. Association of the preoperative total WBC, neutrophils, lymphocytes, and NLR with the clinicopathological characteristics of PTC is depicted in Table 2.

NLR values did not differ in conjunction with gender or age (<45 or ≥45 years). Although we observed a tendency towards higher NLR in larger tumours (especially >4.1 cm), this difference did not reach statistical significance. NLR was also not significantly different, when microcarcinomas were compared to macro-carcinomas. On the other hand, multifocal and bilateral PTC showed higher NLR (2.65 ± 1.08 versus 2.29 ± 0.96, *p* = 0.01 and 2.67 ± 1.15 versus 2.35 ± 0.96, *p* = 0.03, resp.), as did tumours with extrathyroidal invasion (2.74 ± 01.24 versus 2.39 ± 0.96, *p* = 0.04) and positive lymph nodes (3.12 ± 1.07 versus 2.41 ± 1.02, *p* = 0.03). Finally, baseline NLR was not associated with PTC subtype, thyroiditis, or TNM stage.

Comparison of the low versus high NLR subgroups (Table 3) showed again that age and sex did not correlate with NLR; however, high NLR values were associated with larger goitres (38.52 versus 26.01 gr, *p* = 0.009). Tumour size, extrathyroidal invasion, multifocality, bilaterality, presence of thyroiditis, PTC variant, and T stage also did not differ between the groups. On the contrary, cases with lymph node metastases were more frequently encountered in the high than in the low NLR group (11.7% versus 2.9%, *p* = 0.03).

## 4. Discussion

The potential association between chronic inflammation and cancer was first suggested by German pathologist Rudolf Virchow more than a century ago [1]. The underlying mechanisms have been extensively investigated during the past decade, and gastrointestinal malignancies are a fine example of this connection (chronic H. pylori gastritis and gastric cancer, chronic viral hepatitis B and C and hepatocellular carcinoma, idiopathic inflammatory bowel disease and colorectal cancer) [9].

In the field of thyroid research, the relationship between chronic lymphocytic thyroiditis and PTC was first proposed by Dailey in 1955 [10]. Indeed, pathologists have long recognised that certain tumours are densely infiltrated by a mixture of macrophages and lymphocytes (cells of the innate and adaptive immune systems, resp.) both within and around the primary tumour [11].

The innate immune system comprises of mechanisms that protect the host in a nonspecific manner and is mediated by natural killer cells, macrophages, dendritic cells, mast cells, and polymorphonuclear leukocytes (neutrophils, basophils, and eosinophils) [11]. Neutrophils are important mediators of the inflammatory response, and blood neutrophilia has long been recognised as a marker of systemic inflammatory response [1]. On the other hand, lymphocytes of the adaptive immune system have evolved to provide a more versatile defense system plus increased protection following reinfection with the same pathogen [11]. Specifically in cases of malignancy, tumour-associated neutrophilia and/or lymphopenia is considered either a paraneoplastic manifestation or a nonspecific response to cancer-related inflammation due to local tissue destruction and cytokine release [11].

At the molecular level, upregulation of the RET/RAS/BRAF/ERK/MAPK pathway, which has the capability to induce both a proinflammatory and a protumourigenic thyroid programme, is a possible mechanism linking chronic thyroiditis with carcinogenesis [9]. Recent research has further suggested that papillary carcinomas harbour a different genetic background according to the association or not with thyroiditis (expression of BRAF*V600E* in cases without and expression of RET/PTC in cases with autoimmunity) [12]. This is also supported by evidence showing that the RET/PTC oncogene can activate a transcriptional proinflammatory programme in normal thyrocytes [13].

This tumour-host interaction, in the form of systemic inflammatory response, is generally not taken into account in most contemporary prognostic systems [3]. However, inflammatory biomarkers (CRP, cytokines, and white cell counts) can be independent prognostic factors in a variety of tumours (oesophageal, gastric, pancreatic, colorectal, mesothelioma, ovarian, renal, and bladder) [2].

Neutrophil-to-lymphocyte ratio has recently emerged as a simple and valid composite marker of systemic inflammatory response [3]. Although it is inexpensive, easily calculated, and readily available, its use in the preoperative assessment and postoperative follow-up of PTC patients remains a matter of debate. To date, studies examining NLR in well-differentiated thyroid cancer were heterogeneous in statistical methodology, varied largely in their sample sizes (41–3364 PTC cases), and produced inconsistent results (Table 4).

In general, higher NLR values were variably associated with larger tumour size [3, 5, 6, 14], multifocality [14], lymph node metastases [14], and higher TNM stage [4, 6, 14], indicating a more aggressive tumour behaviour and more advanced disease stage. Our study confirmed this relation of high NLR with poor tumour profile, in terms of extrathyroidal invasion, multifocality, bilaterality, and lymph node metastasis.

On the contrary, advanced age (≥45 years) has been previously correlated both with lower [15] and with higher [4, 6] NLR values. In our series, PTC cases ≥ 45 years had a slightly lower NLR (2.39 ± 1.01) compared to <45 years (2.58 ± 1.07), the difference however was not significant (*p* = 0.15).

Interestingly, the only study demonstrating a statistically significant correlation between NLR and the presence of chronic Hashimoto thyroiditis found only a subtle difference in NLR values (1.52 with versus 1.58 without Hashimoto), obviously of minimal clinical importance [15]. Liu et al. and Kocer et al. both showed a trend towards lower NLR values in PTC cases with concurrent chronic lymphocytic thyroiditis [3, 16]. Indeed, several studies have reported an increased incidence of well-differentiated thyroid cancer in patients with thyroiditis, although these patients paradoxically seem to have a better prognosis [17, 18]. Our series could not establish an association between NLR and Hashimoto thyroiditis (NLR 2.48 ± 1.03 with versus 2.36 ± 0.96 without thyroiditis, *p* = 0.59).

An interesting point, confirmed also in this study, is the relatively low NLR values of papillary thyroid cancer, in comparison to other solid tumours. A comprehensive meta-analysis, investigating the association of NLR with patient prognosis in a variety of neoplasms (gastrointestinal, gynaecological, urological, and pulmonary; head and neck, brain, and breast), found indeed NLR medians up to 7.7 [19]. Ratios above the cutoff correlated with unfavourable outcome and worse prognosis, in terms of overall survival (median NLR cutoff 4, range 1.9–7.2), cancer-specific survival (median NLR cutoff 3.85, range 1.9–5.0), progression-free survival (median NLR cutoff 3, range 2.0–5.0), and disease-free survival (median NLR cutoff 5.0, range 2.0–7.7). Our cohort exhibited a mean NLR of 2.44 ± 1.02 and a median NLR of 2.17 (range 0.86–6.14). Furthermore, only 12 patients (5.9%) presented with ratios greater than 4. Similar results have been obtained in all studies dealing with NLR in well-differentiated thyroid cancer (Table 5). Even in anaplastic carcinomas, which represent the most aggressive form of thyroid neoplasia, a diagnostic cutoff of 3.8 is proposed, to aid discrimination against well-differentiated cancers [8].

This phenomenon has been attributed to the fact that inflammation may be less important in the initiation of thyroid carcinogenesis and to the relatively indolent nature of differentiated thyroid cancer, which causes a less vigorous systemic inflammatory response [3]. In any case, NLR remains largely a nonspecific biomarker of systemic inflammation. Although promising in terms of sensitivity, careful exclusion of all patients with medical conditions affecting white blood cell differential counts (acute infections, allergic reactions, cardiovascular incidents etc.) is mandatory. Nevertheless, the NLR is universally available from routine blood tests and does not increase the preoperative diagnostic work-up cost [2].

Limitations of our study protocol include its single-institution, retrospective nature and average sample size. Furthermore, our study could not incorporate the most recent changes in terminology of the follicular variant of PTC [20]. The subgroup “follicular variant” was inevitably heterogeneous and included both cases of infiltrative and encapsulated follicular variant PTC. Modern molecular profiling however has indicated that the latter closely resembles follicular adenoma/follicular carcinoma, whereas the former behaves like the classic PTC. Indeed noninvasive, encapsulated follicular variant of PTC has been reclassified as “Noninvasive follicular thyroid neoplasm with papillary-like nuclear features” [20]. We therefore consider that such retrospective comparison between classic and follicular variants of PTC may probably be subject to bias and inaccuracy.

Finally, our study protocol was neither designed to address the issue of NLR in conjunction with patient prognosis nor to propose specific cutoff values. Given the generally excellent 5-year and 10-year disease-free and overall survival of PTC patients, a long-term follow-up would be required to draw safe, meaningful conclusions. Only 3 studies have reported long-term patient outcomes, with a mean follow-up of 60 months [5, 6, 8]. While Kim et al. showed worse disease-free survival in stage III-IV patients with an NLR cutoff of 1.5, Lang et al. and Cho et al. found that NLR determined neither disease-free survival nor cancer-specific death rates [5, 6, 8]. On the other hand, the association of higher NLR with aggressive clinical and pathological characteristics may imply unfavourable outcome. Our aim is to closely monitor these patients over the following years, in order to assess the prognostic value of NLR in PTC cases.

Implications for future study include also investigating the possible association between intra- and peritumoural immune cell infiltration with systemic white blood cell counts, as well as the expected normalisation of postthyroidectomy levels of the NLR.

## 5. Conclusion

Whether increased NLR in more aggressive tumours represents the inflammatory microenvironment leading to tumourigenesis, or is a tumour-associated phenomenon, remains to be elucidated. Whereas NLR was significantly increased in invasive, bilateral, multifocal, and lymph node-positive papillary cancers, our study found no difference in NLR values between patients with and without chronic lymphocytic thyroiditis. As a surrogate systemic inflammatory biomarker, NLR is inexpensive, readily available, and warrants further study in larger trials.

## Figures and Tables

**Table 1:** Demographic characteristics and haematological data of PTC patients.

*N*	205	
Sex		
Male	55	26.8%
Female	150	73.2%
Age (years)	51.2 ± 14.7	Range 18–89
<45 years	65	31.7%
≥45 years	140	68.3%
Thyroid specimen weight (gr)	32.7 ± 37.46	Range 5–329
TSH (mIU/L)	1.45 ± 1.48	Range 0.29–4.19
WBC total (cells/mL)	7527 ± 2166	Range 4200–10,000
Neutrophils (cells/mL)	4744 ± 1767	Range 1900–9100
Lymphocytes (cells/mL)	2084 ± 661	Range 900–5200
NLR (mean ± SD)	2.44 ± 1.02	Range 0.86–6.14

**Table 2:** Association of preoperative total WBC, neutrophils, lymphocytes, and NLR with clinicopathological characteristics of PTC.

	*N*	Total WBC	*p* value	Neutro	*p* value	Lympho	*p* value	NLR	*p* value
Sex									
Male	55	7682 ± 2131		4868 ± 1787		2034 ± 723		2.62 ± 1.19	
Female	150	7495 ± 2179	0.31	4714 ± 1765	0.31	2111 ± 637	0.26	2.38 ± 0.96	0.09
Age									
<45 years	65	7857 ± 2637		5070 ± 2268		2053 ± 602		2.58 ± 1.07	
≥45 years	140	7419 ± 1934	0.12	4628 ± 1510	0.07	2105 ± 684	0.33	2.39 ± 1.01	0.15
Tumour size									
0.1–1 cm	113	7421 ± 2161		4686 ± 1850		2051 ± 558		2.40 ± 0.96	
1.1-2 cm	57	7682 ± 2332		4741 ± 1639		2277 ± 879		2.32 ± 1.10	
2.1–4 cm	29	7880 ± 2105		5020 ± 1822		2060 ± 588		2.61 ± 1.16	
>4.1 cm	6	7600 ± 935	0.81	5200 ± 752	0.82	1540 ± 518	0.07	3.55 ± 0.73	0.12
Tumour size									
0.1–1 cm	113	7421 ± 2161		4686 ± 1850		2051 ± 558		2.40 ± 0.96	
>1.1 cm	92	7738 ± 2164	0.18	4864 ± 1638	0.27	2152 ± 793	0.17	2.51 ± 1.13	0.26
Extrathyroidal invasion									
Yes	43	7438 ± 1987		4779 ± 1753		1897 ± 569		2.74 ± 1.24	
No	162	7548 ± 2208	0.4	4744 ± 1774	0.46	2118 ± 675	0.05	2.39 ± 0.96	**0.04**
Multifocality									
Yes	91	7684 ± 2259		4983 ± 1920		1999 ± 623		2.65 ± 1.08	
No	114	7444 ± 2093	0.24	4589 ± 1636	0.08	2157 ± 681	0.06	2.29 ± 0.96	**0.01**
Bilaterality									
Yes	65	7860 ± 2337		5127 ± 2039		2042 ± 621		2.67 ± 1.15	
No	140	7413 ± 2080	**<0.01**	4601 ± 1625	**0.04**	2110 ± 677	0.27	2.35 ± 0.96	**0.03**
Lymph node metastasis									
Yes	15	7050 ± 1089		4800 ± 1235		1613 ± 331		3.12 ± 1.07	
No	190	7570 ± 2201	0.25	4754 ± 1793	0.47	2115 ± 664	**0.02**	2.41 ± 1.02	**0.03**
Histologic subtype									
Classic	70	7450 ± 1986		4658 ± 1597		2069 ± 608		2.41 ± 0.97	
Follicular variant	135	7607 ± 2275	0.33	4819 ± 1874	0.29	2104 ± 694	0.37	2.47 ± 1.07	0.36
Thyroiditis									
No	88	7297 ± 2093		4538 ± 1662		2049 ± 608		2.36 ± 0.98	
Nonspecific	90	7976 ± 2167		5113 ± 1892		2173 ± 686		2.54 ± 1.10	
Hashimoto	27	7226 ± 2279	0.14	4530 ± 1681	0.13	2008 ± 760	0.45	2.48 ± 1.03	0.59
T stage (AJCC)									
T1a	107	7407 ± 2168		4666 ± 1849		2055 ± 559		2.38 ± 0.95	
T1b	37	8027 ± 2545		4977 ± 1731		2381 ± 965		2.32 ± 1.02	
T2	17	7780 ± 1708		4890 ± 1194		2150 ± 700		2.44 ± 0.86	
T3	44	7500 ± 1829	0.61	4814 ± 1731	0.86	1928 ± 582	0.07	2.76 ± 1.30	0.34
TNM stage (AJCC)									
I	169	7588 ± 2268		4474 ± 1861		2121 ± 664		2.40 ± 1.00	
II	10	7920 ± 944		5440 ± 573		1760 ± 297		3.14 ± 0.48	
III	21	7073 ± 1642		4340 ± 1134		1967 ± 704		2.50 ± 1.20	
IVa	5	6675 ± 645	0.67	4600 ± 868	0.66	1400 ± 163	0.09	3.37 ± 1.05	0.11

The bold data represent statistically significant *p* values.

**Table 3:** Comparison of prevalence of prognostic factors in the low and high NLR subgroups.

	Low NLR (<2.17)	High NLR (>2.17)	*p* value
*N*	102	103	
Sex			
Male	25 (24.5%)	30 (29.1%)	
Female	77 (75.5%)	73 (70.9%)	0.46
Age (years)	52.74 ± 13.18	51.15 ± 15.45	0.24
Specimen weight (gr)	26.01 ± 18.24	38.52 ± 43.39	**0.009**
Tumour size (cm)	0.95 ± 0.69	1.30 ± 1.43	0.38
Extrathyroidal invasion	20 (19.6%)	23 (22.3%)	0.63
Lymph node metastasis	3 (2.9%)	12 (11.7%)	**0.03**
Multifocality	43 (42.1%)	48 (46.6%)	0.52
Bilaterality	30 (29.4%)	35 (33.9%)	0.48
Thyroiditis	52 (50.9%)	65 (63.1%)	0.08
Follicular variant	68 (66.7%)	67 (65%)	0.81
T stage			
T1a + T1b	75 (73.5%)	69 (67%)	
T2 + T3	27 (26.5%)	34 (33%)	0.31

The bold data represent statistically significant *p* values.

**Table 4:** Studies examining NLR in association with clinicopathological characteristics of PTC.

	Association found	No association found
Age	Liu et al. [4] (lower NLR in patients < 45 years) Lang et al. [6] (lower NLR in patients < 45 years) Kim et al. [15] (lower NLR in patients ≥ 45 years)	Liu et al. [3] Kim et al. [5] Gong et al. [14] Manatakis et al.

Sex		Liu et al. [3], Liu et al. [4], Kim et al. [5], Lang et al. [6], Gong et al. [14] Manatakis et al.

Tumour size	Liu et al. [3], Kim et al. [5], Lang et al. [6], Gong et al. [14]	Liu et al. [4], Cho et al. [8], Kim et al. [15], Manatakis et al.

Extrathyroidal invasion	Manatakis et al.	Liu et al. [3], Liu [4] Kim et al. [5], Lang et al. [6], Kim et al. [15]

Multifocality	Gong et al. [14], Manatakis et al.	Liu et al. [3], Kim et al. [5], Lang et al. [6], Kim et al. [15]

Bilaterality	Manatakis et al.	Lang et al. [6]

Lymphovascular invasion		Liu et al. [3], Lang et al. [6], Kim et al. [15]

Lymph node metastasis	Gong et al. [14], Manatakis et al.	Liu et al. [3], Liu et al. [4], Kim et al. [5], Lang et al. [6], Kim et al. [15]

TNM stage	Liu et al. [4] (stages I-II versus III-IV) Lang et al. [6] (for stage I) Gong et al. [14] (for patients ≥ 45 years)	Liu et al. [3] Kim et al. [5] Kim et al. [15] Manatakis et al.

Histologic subtype		Lang et al. [6], Manatakis et al.

Thyroiditis	Kim et al. [15] (lower NLR in Hashimoto)	Liu et al. [3], Lang et al. [6], Kocer et al. [16], Manatakis et al.

Recurrence risk	Liu et al. [3]	

Prognosis	Kim et al. [5] (for stages III-IV)	Lang et al. [6], Cho et al. [8]

**Table 5:** Mean or median NLR values in studies on PTC (N/R not reported).

Name	Year	Number of PTC cases	NLR (mean or median)	Range
Liu et al. [3]	2012	159	1.94	1.50–2.57
Seretis et al. [2]	2013	52	3 in PTMC 3.4 in PTC	N/R
Kim et al. [5]	2013	542	1.74	N/R
Lang et al. [6]	2014	191	2.68	0.52–16.6
Liu et al. [4]	2015	321	1.99 in <45 years 2.28 in >45 years	0.4–8.58
Cho et al. [8]	2015	3364	1.86	0.28–16.29
Kocer et al. [16]	2015	65	2.47 with thyroiditis 2.57 without thyroiditis	N/R
Kim et al. [15]	2015	1066	1.75 in <45 years 1.52 in ≥45 years	0.25–10.20
Gong et al. [14]	2016	161	2	0.64–6.38
Yaylaci et al. [7]	2016	41	1.9	N/R
Manatakis et al.		205	2.44	0.86–6.14
